# Effectiveness of Psychological Therapy for Treatment-Resistant Depression in Adults: A Systematic Review and Meta-Analysis

**DOI:** 10.3390/jpm15080338

**Published:** 2025-08-01

**Authors:** Sabrina Giguère, Alexandra Fortier, Julie Azrak, Charles-Édouard Giguère, Stéphane Potvin, Alexandre Dumais

**Affiliations:** 1 Department of Psychiatry and Addictology, University of Montreal, Montreal, QC H3T 1J4, Canada; sabrina.giguere.2@umontreal.ca (S.G.); julie.azrak@umontreal.ca (J.A.); stephane.potvin@umontreal.ca (S.P.); 2 Research Center of the University Institute in Mental Health of Montreal, Montreal, QC H1N 3V2, Canada; cedouard-giguere.iusmm@ssss.gouv.qc.ca; 3 Services et Recherches Psychiatriques AD, Montreal, QC H1N 3V2, Canada; 4 Institut National de Psychiatrie Légale Philippe-Pinel, Montreal, QC H1C 1H1, Canada

**Keywords:** systematic review, meta-analysis, major depressive disorder, treatment-resistant, psychotherapy

## Abstract

**Background:** Depression that is resistant to two or more adequate treatment trials—treatment-resistant depression (TRD)—is a prevalent clinical challenge. Although psychotherapies have been recommended by clinical guidelines as an alternative or adjunctive treatment strategy, the effectiveness of psychotherapy in individuals with TRD has not yet been evaluated through meta-analytic methods, primarily due to a limited number of trials. This highlights the necessity of personalized research targeting this specific population. This systematic review and meta-analysis aimed to summarize the evidence on psychotherapy in treating TRD. **Methods:** A systematic search was conducted following the Guidelines from Preferred Reporting Items for Systematic Reviews and Meta-Analyses (PRISMA). Articles were included if they quantitatively examined the efficacy of psychotherapy on depression symptoms in individuals diagnosed with depression who had not responded to at least two prior treatments (i.e., pharmacotherapy and/or psychotherapy). **Results:** A total of 12 studies were included. The quality of evidence was evaluated as being globally moderate. When pooling all psychotherapies, a small-to-moderate, but significant, effect on depressive symptoms was observed compared to the control group (SMD = −0.49, CI = −0.63; −0.34). The observed effect remained unchanged after removing the outlier (SMD = −0.47, CI = −0.62; −0.32). When examining depressive symptoms by type of psychotherapy, Mindfulness-Based Cognitive Therapy (SMD = −0.51, CI = −0.76; −0.25), Cognitive Behavioral Therapy (SMD = −0.53, CI = −0.92; −0.14), and Cognitive Therapy (SMD = −0.51, CI = −1.01; −0.01) showed a moderately significant effect on depressive symptoms compared to the control group. **Conclusions:** Although this potentially represents the first meta-analysis in this area, the number of studies specifically addressing this complex population remains limited, and the existing literature is still in its early stages. Research focusing on TRD is notably sparse compared to the broader body of work on depression without treatment resistance. Consequently, it was not possible to conduct meta-analyses by type of psychotherapy across all treatment modalities and by type of control group. Due to several study limitations, there is currently limited evidence available about the effectiveness of psychotherapy for TRD, and further trials are needed. Beyond the treatments usually offered for depression, it is possible that TRD requires a personalized medicine approach.

## 1. Introduction

The lifetime prevalence of major depressive disorder (MDD) is up to 21% and tends to increase over the years [1,2]. MDD can impact all areas of life, including professional, relational, and personal spheres [3,4]. For example, MDD is associated with a prevalence of up to 30% for work absenteeism or unemployment [5]. It also increases the risk of developing medical issues, such as cardiovascular and respiratory diseases [6]. Additionally, the lifetime prevalence of suicide attempts in MDD was estimated at 31% (95% confidence interval (CI) = 27–34%) [7]. Indeed, the risk of death by suicide was significantly higher among individuals with MDD, with a relative risk of 19.7 (CI = 12.2–32.0) compared to individuals without MDD [8]. In 2023, the societal economic burden of MDD in the United States of America was estimated to be $382.4 billion [9]. Furthermore, the World Health Organization estimates that depression is among the leading causes of disability in developed countries [10].

Treatments recommended for depression are antidepressants, psychotherapy, and their combination [11,12]. However, resistance to antidepressant treatment is a common issue in clinical practice. Studies indicate that between 30% and 55% of individuals remain symptomatic after two trials of antidepressants [13,14,15]. Treatment resistance in MDD leads to more severe consequences across various aspects of individuals’ lives [15]. Individuals with treatment-resistant depression (TRD) experience poorer quality of life and psychosocial functioning [16,17]. They are twice as likely to attempt or complete suicide and have higher rates of hospitalization [18,19]. They also use more healthcare resources and impose a greater societal and economic burden than those with treatment-responsive depression [20,21]. Furthermore, increasing levels of TRD were associated with elevated direct and indirect costs, as well as a decline in health-related quality of life [22]. Given the high prevalence of individuals with TRD and the negative repercussions for individuals, their entourage, and society, psychotherapy has been widely recommended in clinical practice guidelines as part of the treatment for those suffering from MDD and TRD [11,12,23,24,25].

Although several meta-analyses have examined the efficacy of psychotherapies for treating depressive symptoms in adults, treatment resistance was rarely considered. In adults without specific criteria for treatment resistance, a large meta-analysis of 143 studies reported that psychotherapy had a moderate effect on depressive symptoms compared to treatment as usual (TAU) (g = −0.66, CI = −0.78; −0.53) [26]. Analyses by type of psychotherapy revealed moderate efficacy: Cognitive Behavioral Therapy (CBT) (k = 94, g = 0.71, CI = 0.62; 0.79), Behavioral Activation Therapy (k = 31, g = 0.74, CI = 0.56; 0.91), Interpersonal Psychotherapy (k = 31, g = 0.60, CI = 0.45, 0.75), Problem-Solving Therapy (k = 13, g = 0.83, CI = 0.45; 1.21), Nondirective Supportive Therapy (k = 18, g = 0.58, CI = 0.45; 0.72), and Short-Term Psychodynamic Psychotherapy (k = 10, g = 0.61, CI = 0.33, 0.88) [27]. Among individuals with resistance to at least one antidepressant treatment, the number of available studies was significantly reduced (k = 6), as was their efficacy [28]. Indeed, CBT combined with TAU has shown a small but statistically significant benefit compared to TAU alone (k = 3, SMD = −0.35, CI = −0.56; −0.13). However, other psychotherapies in addition to TAU, such as Dialectical Behavior Therapy (k = 1, SMD = −0.72, CI = −1.66; 0.21), Interpersonal Therapy (k = 1, SMD = 0.07, CI = −0.60; 0.74), and Intensive Short-Term Dynamic Psychotherapy (k = 1, SMD = −0.88, CI = −1.41; −0.35) have not shown significant effects compared to TAU alone. To our knowledge, no meta-analysis has specifically focused on individuals who remain symptomatic after two adequate treatment courses. Previous attempts to conduct such meta-analyses have been limited by the small number of eligible studies [28,29]. Notably, a recent meta-analysis identified only three studies, which were three different types of psychotherapy: CBT, Long-Term Psychoanalytic Psychotherapy, and Mindfulness-Based Cognitive Therapy (MBCT). Moreover, outcomes were limited to depression remission, with no data reported on secondary outcomes such as self-esteem, anxiety, functioning, or quality of life [28]. Therefore, personalized research targeting this specific population was necessary.

For instance, systematic reviews that defined treatment resistance as non-remittent depression despite at least two antidepressant trials identified only three relevant studies [28,29]. In addition, a recent article from 2023 highlighted the issues regarding the multiple definitions of TRD and reported the need to include non-response to psychotherapies in the definition of treatment resistance in MDD [15]. Incorporating non-response to psychotherapy into the criteria for treatment resistance aligns with the established clinical guidelines for treating depression (i.e., American Psychological Association, National Institute for Health and Care Excellence, Canadian Network for Mood and Anxiety Treatments). These guidelines recommend psychotherapy as a first-line treatment, as well as an intervention for cases where patients do not respond or only partially respond to pharmacotherapy [11,12,25]. Moreover, the inclusion of non-response to psychotherapy in the definition of treatment resistance facilitates the use of real-world clinical samples, as approximately 25% of treatment modalities in clinical practice involve psychotherapy [30].

Thus, the present systematic review aimed to evaluate the effectiveness of all types of psychotherapy, both combined and separately, in individuals with TRD. All psychotherapies were initially pooled to provide an overall estimate of their effectiveness and to enhance statistical power before individual modalities were analyzed. The outcomes assessed extended beyond depressive symptoms to include associated domains such as anxiety, self-esteem, overall functioning, and quality of life. Consistently with the definitions discussed above, TRD was defined as non-response to at least two treatment courses, including pharmacotherapy and/or psychotherapy. Given that treatment effectiveness tends to decline with increasing resistance across various psychopathologies, the efficacy of psychotherapy was expected to be lower in TRD compared to non-resistant MDD.

## 2. Methods

### 2.1. Search Strategy

The search was performed in accordance with the Preferred Reported Items for Systematic Reviews and Meta-Analyses (PRISMA) guidelines [31] (see Table S1 in Supplementary Materials). Two graduate students (A.F. and S.G.) independently conducted a systematic search of the electronic databases PubMed (k = 3550), PsycINFO (k = 1208), Web of Science (k = 3120), and Cochrane Library (k = 264). The search included records from the inception of the databases until October 2024. Search terms were inclusive of depressive disorder (e.g., major depressive disorder, depression), treatment-resistance (e.g., refractor, nonresponse, resistant), and psychological therapy (e.g., psychotherapy, intervention, psychosocial treatment). No restrictions were applied to the setting, date, or geographical location. English and French language sources were eligible. A secondary search was conducted in Google Scholar to retrieve gray literature, and the reference lists of the included studies (k = 38) were screened to ensure that no relevant studies were missed. The specific search strategy adapted for each database is provided in Table S2 in the Supplementary Materials. The systematic review was not preregistered.

### 2.2. Study Eligibility

Studies were included for analyses if they met the following criteria: (1) quantitatively examined the effects of psychological therapy on depressive symptoms, (2) involved adults with depression, (3) examined treatment resistance—defined as unremitted depression despite at least two courses of treatment (i.e., pharmacotherapy and/or psychotherapy), (4) clearly involved a healthcare professional in the psychotherapy, and (5) compared psychotherapy to a control group (e.g., other psychotherapy, waiting list, TAU, pharmacotherapy). To maximize the number of studies and obtain an overall view on the subject, quasi-experimental studies were included in addition to clinical trials (e.g., randomized controlled trials (RCTs)). Studies were excluded if they (1) did not involve a healthcare professional in the psychotherapy (e.g., self-help, computer-based), (2) merged multiple types of psychotherapy into a single outcome category (e.g., reporting results for all psychotherapies combined), (3) were not peer-reviewed, (4) comprised pharmacotherapy alone without psychotherapies, and (5) involved prevention programs. Self-help and computer-based interventions were excluded, because they are not included in the recommendations of clinical guidelines for the treatment of depression [11,12,25]. Study eligibility was conducted independently by A.F., S.G., and J.A., and discussions on including meta-analyses were held with senior researchers (A.D. and S.P.) to ensure consensus.

### 2.3. Data Extraction

Data were extracted with a standardized form by S.G. and counter-validated by J.A. Key information related to the design of studies, types of psychosocial treatment, country of study, participants (i.e., definition of treatment resistance, sample size, gender), control group (e.g., TAU, other psychotherapy), timepoint (i.e., post-therapy, 3-month follow-up), outcomes measured (e.g., depressive and related symptoms, functioning, quality of life), study results, and adjustment for confounding factors were recorded (see Table S3 in the Supplementary Materials). The authors were contacted when data were missing to perform our analyses or when the condition of a group was unclear. In the absence of a response from the authors, their results were not included in the analysis. Furthermore, S.G. and J.A. independently assessed the quality of evidence for the effect sizes reported in the meta-analyses using a set of criteria based on the GRADE Checklist [32,33,34]. This widely used and recommended tool enables the evaluation of key domains of evidence quality, including risk of bias, indirectness, inconsistency, imprecision, and publication bias [35,36,37,38]. We assigned higher scores to studies that comprised a single-blind RCT, comprised larger sample sizes compared to active control (i.e., another psychotherapy), and conducted moderator analyses (e.g., number of treatment sessions, type of comparison condition, demographic predictors). Studies were assigned to the following categories: very low quality, low, moderate-to-low, moderate, moderate-to-high, and high.

### 2.4. Statistical Analysis

The analyses were performed using the statistical software RStudio (version 2024.12.1) with the metafor package [39]. The effect size of depressive symptoms was estimated with standardized mean differences (SMDs). For all studies, CIs were calculated based on post-treatment scores comparing the experimental group to the control group. When the CI crosses zero, it indicates that the effect was not statistically significant. Random-effects models were employed, which are more conservative than fixed-effects models and appear to address heterogeneity between studies and study samples [40]. The following qualitative descriptions of the strength of reported SMDs were used: small 0.2, medium 0.5, and large ≥ 0.8 [41]. When a study evaluated the effects of depression using clinician-rated and self-report measures, both were used, and a multivariate random-effect meta-analysis with a random intercept grouped by study was fitted to the data. A meta-analysis suggested that combining self-report and clinician ratings may provide a more accurate assessment of treatment response, as different symptoms may be more suitable for self-report or ratings by clinicians [42]. Heterogeneity among study point estimates was quantified using the I^2^ index, with a value of 0% indicating no effect heterogeneity. Values of 25%, 50%, and 75% correspond to low, moderate, and high heterogeneities, respectively [43]. The risk of publication bias was assessed using Egger’s test and examined with funnel plots. The Egger’s test is widely used in meta-analyses of psychotherapy [36,44,45]. A significant *p*-value suggests the presence of publication bias, indicating that studies with non-significant results are more likely to remain unpublished [46]. Additional analyses were also performed by type of psychotherapy. Due to the limited number of studies, all analyses were conducted irrespective of control group type. Sub-analyses were conducted without the outlier study. Analyses were performed by S.G. and validated by a statistician (C.-É.G.).

## 3. Results

### 3.1. Description of Studies Included

**Figure 1:**
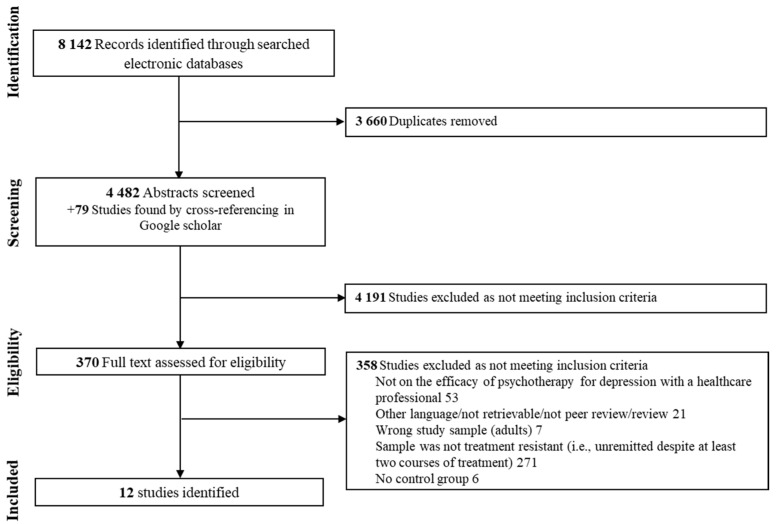
Flow chart of the study selection process.

The PRISMA flowchart for study inclusion is presented in Figure 1. The systematic search identified 4561 potential articles, which were screened for eligibility after duplicates were removed. Among these, twelve studies were included, comprising a total of 723 adults with depression and a non-response to at least two treatment courses (65% women, mean age = 43.87, SD = 2.68). Eleven of these studies reported the presence of psychiatric comorbidities (e.g., anxiety disorder, personality disorder, substance use disorder). Moreover, eleven studies were randomized, seven controlled, and eight had evaluators blinded to the type of intervention. Psychotherapy comprised MBCT (k = 3), CBT (k = 2), cognitive therapy (k = 2), long-term psychoanalytic psychotherapy (k = 1), group compassion-focused therapy (k = 1), trauma-focused cognitive behavioral therapy (k = 1), group-based interpersonal psychotherapy and occupational therapy (k = 1), and body-oriented psychological therapy (k = 1). Control groups varied across studies, including TAU (k = 6), eye movement desensitization and reprocessing (k = 1), waiting list (k = 1), CBT combined with rehabilitation treatment (k = 1), rehabilitation treatment alone (k = 1), pharmacotherapy (k = 2), and a health-enhancement program (k = 1). Studies have been carried out in various countries (Canada (k = 1), England (k = 1), Iceland (k = 1), Iran (k = 1), Italy (k = 1), Japan (k = 1), Netherlands (k = 1), United Kingdom (k = 2), United States of America (k = 3)). Findings were mostly evaluated as having moderate quality evidence. See Table S3 in Supplementary Materials for a summary of the quality of evidence provided by the included studies. The study by Foroughi et al., 2020 [47] was found to be an outlier since they were two standard deviations below or above the composite effect size.

### 3.2. Effects of Psychological Interventions

**Figure 2:**
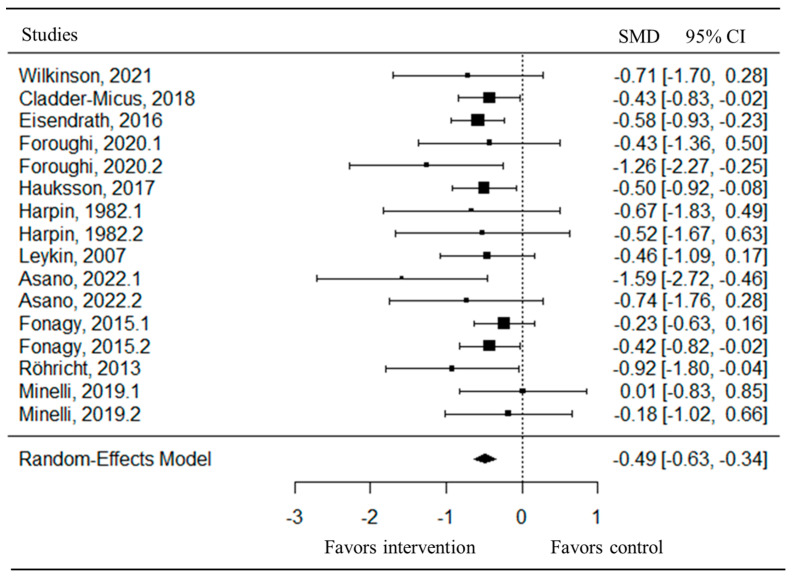
Forest plot of the effect size of the psychotherapy compared to the control group on depressive symptoms [47,48,49,50,51,52,53,54,55,56,57].

**Figure 3:**
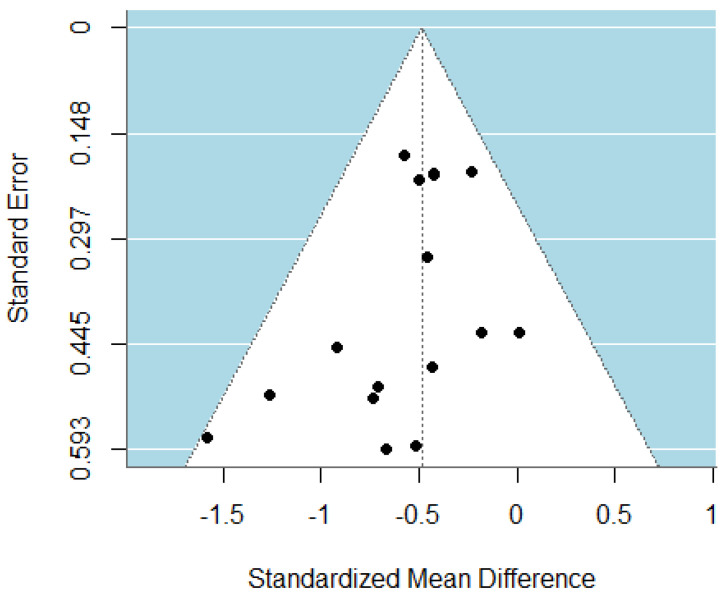
Funnel plot of the meta-analysis of psychotherapy for TRD.

The pooled analysis of all studies showed a small-to-moderate, but significant, post-intervention effect on depressive symptoms (SMD = −0.49, CI = −0.63; −0.34) in favor of the psychotherapy compared to the control group (Figure 2). There was no heterogeneity (I^2^ = 0.00%) between studies. Upon examination of the funnel plot, no publication bias was observed (Figure 3). Egger’s test also suggested that there was no publication bias for the overall database (t = −1.69, *p* = 0.11). Some of these studies assessed depressive symptoms with self-reports, involving clinical judgment, or using both questionnaires. After removing the outlier study [47], the effect on depressive symptoms remaining stayed stable (SMD = −0.47, CI = −0.62; −0.32).

### 3.3. Effects of Psychological Interventions by Type of Intervention

#### 3.3.1. Mindfulness-Based Cognitive Therapy

An RCT compared eight sessions of MBCT, delivered in groups of 8–12 participants in addition to TAU (*n* = 44), with TAU alone (*n* = 52) [48]. Participants were included if their current depressive episode had lasted at least 12 months, had moderate-to-high levels of depressive symptoms, and had at least one trial of antidepressant (appropriate dose for ≥four weeks) and a previous CBT or interpersonal therapy (≥10 sessions) during the current depressive episode. The TAU condition was a naturalistic condition consisting of mental healthcare for depression. It included antidepressant medication, psychological treatment excluding mindfulness-based treatment, support by a psychiatric nurse, and day hospital treatment. At post-therapy, a small but significant improvement was observed in depressive symptoms measured using the Inventory of Depressive Symptomatology-Self Report (SMD = −0.43, CI = −0.83; −0.02) and in quality of life (SMD = 0.45, CI = 0.03; 0.88), favoring MBCT plus TAU. Additionally, moderate improvements were found in mindfulness skills (SMD = 0.67, CI = 0.24; 1.10) and self-compassion (SMD = 0.52, CI = 0.09; 0.95). There was no significant difference between groups in rumination (SMD = −0.38, CI = −0.80; 0.05). Apart from higher baseline rumination, which was associated with a greater reduction in depressive symptoms in the MBCT plus TAU condition compared to TAU alone, most moderators had no significant treatment effect (e.g., sex, age, childhood trauma, number of previous episodes, duration of the current episode, treatment resistance, baseline depression severity). The authors reported no difference between groups in the mean number of treatment sessions or in the number of patients who received TAU. The quality of evidence is graded as moderate.

A second large RCT of eight sessions of MBCT delivered in groups of 6–12 individuals (*n* = 67) was compared to a health-enhancement program (*n* = 64), which included behavioral activation [49]. Participants included had a diagnosis of MDD, had a score of 14 or greater on the 17-Hamilton Depression Rating Scale (HDRS), and had undergone two or more adequate antidepressant medication trials during the current episode. At post-therapy, a moderate reduction was found in depressive symptoms measured using the HDRS (SMD = −0.58, CI = −0.93; −0.23) in favor of MBCT. The MBCT showed no significant difference in mindfulness skills (SMD = 0.15, CI = −0.01; 0.30), rumination (SMD = 0.05, CI = −0.39; 0.29), and experiential avoidance (SMD = 0.00, CI = −0.34, 0.34) compared to the health-enhancement program group. In both groups, participants were allowed to make medication changes during treatment, but no significant difference was found between the groups for this variable. The moderators, such as anxiety, presence of a personality disorder, and emotional abuse in childhood, had a significantly higher depressive symptom severity. The quality of evidence was graded as moderate.

A randomized trial compared eight sessions of MBCT in addition to receiving antidepressants (*n* = 9) to receiving antidepressants alone (*n* = 9) (i.e., 60 mg citalopram/4–6 weeks, 200 mg sertraline/4–6 weeks plus bupropion (dose and duration not mentioned)) [47]. The participants had a diagnosis of MDD, a minimum of a moderate level of depression, and were resistant to at least two antidepressants. After therapy, compared to the control group, participants receiving MBCT combined with antidepressants showed a large improvement in depressive symptoms measured using the Beck’s Depression Inventory II (BDI-II) (SMD = −1.26, CI= −2.27; −0.25), but no significant change was observed when symptoms were assessed using the HDRS (SMD = −0.43, CI = −1.36, 0.50). However, at the 1-month follow-up, a significant difference between groups was found for depressive symptoms assessed with the HDRS (SMD = −2.45, CI= −3.67; −1.23), but not when assessed using the BDI-II (SMD = −0.91, CI = −1.88; 0.06). In addition, post-therapy, the MBCT group showed significant improvements in rumination (SMD = −1.06, CI = −2.04; −0.07) and mindfulness skills (SMD = 2.16, CI: 1.00; 3.32) compared to the control group. These improvements were maintained at the 1-month follow-up. Finally, no significant change in self-compassion was observed after therapy (SMD = 1.14, CI = −0.57; 2.86). However, a large improvement in favor of the MBCT group was found at the 1-month follow-up (SMD = 3.09, CI = 1.73; 4.46). The quality of evidence was graded as low.

At post-therapy, the pooled analysis of these three studies, including 245 participants, showed a moderate improvement in depressive symptoms (SMD = −0.55, CI = −0.80; −0.31, I^2^ = 0.00%) and a small improvement in mindfulness skills (SMD = 0.30, CI = −0.05; 0.55, I^2^ = 63.27%) in favor of MBCT compared to the control group. However, there was no significant difference in rumination (SMD = −0.32, CI = −0.72; 0.09, I^2^ = 49.18%) and self-compassion (SMD = 1.14, CI = −0.57; 2.86, I^2^ = 96.79%) when compared to an active control group. The analysis excluding the outlier study, which included two studies totaling 227 participants, showed that the effect remained unchanged in depressive symptoms (SMD = −0.51, CI = −0.78; −0.25, I^2^ = 0.00%), mindfulness skills (SMD = 0.22, CI = 0.03; 0.40, I^2^ = 36.21%), rumination (SMD = −0.19, CI = −0.50; 0.12, I^2^ = 26.71%), and self-compassion (SMD = 0.31, CI = −0.06; 0.68, I^2^ = 44.96%) in favor of MBCT compared to the control group. However, the heterogeneity between studies was reduced by removing the outlier study.

#### 3.3.2. Cognitive Behavioral Therapy

A large randomized trial compared 12 sessions of individual CBT (*n* = 59) and group CBT (*n* = 86; groups of 12–15)—which both included rehabilitation treatment—and rehabilitation treatment alone (*n* = 36) [50]. Participants were inpatients diagnosed with MDD (83.9%) or dysthymia (36.5%) and failed to respond to at least two antidepressant trials of adequate doses and duration. The rehabilitation treatment consisted of psychoeducation, behavioral activation, occupational therapy, relaxation, counseling by a member of the professional health or social care, and medication as needed. Most participants received high doses of antidepressants on arrival, and the medication was continued throughout the study. At post-therapy, a moderate improvement in depressive symptoms was observed on the BDI-II (SMD = −0.50, CI = −0.92; −0.08) and hopelessness (SMD = −0.53, CI = −0.95; −0.11) in favor of individual CBT plus rehabilitation treatment compared to rehabilitation treatment alone. However, these benefits were not maintained at the 18-month follow-up (SMD = −0.14, CI = −0.66; 0.38; SMD = −0.36, CI = −0.88; 0.17). There was no difference between these two groups in anxiety (SMD = −0.17, CI = −0.59; 0.24) and automatic thoughts (SMD = −0.31, CI = −0.72; 0.11), at either post-therapy or 18-month follow-up. No significant difference was found between group CBT plus rehabilitation treatment and rehabilitation treatment alone, or between individual CBT plus rehabilitation treatment and group CBT plus rehabilitation treatment, at either post-therapy or 18-month follow-up. Most comorbidities (i.e., generalized anxiety disorder, panic disorder, post-traumatic stress disorder, hypomanic episode, obsessive–compulsive disorder, psychotic disorder, bulimia, and anorexia) showed no significant impact on treatment outcomes, except for social phobia and substance dependence, which were associated with worse depression severity after the therapy. The quality of evidence ranged from low-to-moderate to moderate-to-high depending on the control group and follow-up duration.

A pilot study began with six ketamine infusions over three weeks, and then the 28 individuals who achieved a clinical response were randomized to 16 sessions of CBT (*n* = 14) or TAU (*n* = 14) [51]. TAU consisted of weekly or every-other-week visits with a study physician for the management of medication and adverse events. The study did not report if there was a difference between groups in medication management. Participants included had a diagnosis of MDD, severe depressive episodes, and were resistant to two or more adequate courses of antidepressants. At post-therapy, no significant difference was found in depressive symptoms measured with the Montgomery-Asberg Depression Rating Scale (MADRS) (SMD = −0.65, CI = −1.82; 0.55) nor with the Quick Inventory of Depressive Symptomatology-Self Report (SMD = −0.71; CI = 0–1.70; 0.28). The quality of evidence was graded as low-to-moderate.

At post-therapy, the pooled analysis of these two studies (*n* = 209) showed a significant moderate reduction in depressive symptoms (SMD = −0.53, CI = −0.92; −0.14) in favor of CBT compared to the control group. There was no heterogeneity between studies (I^2^ = 0.00%).

#### 3.3.3. Cognitive Therapy

An RCT was conducted comparing 20 to 28 sessions of Cognitive Therapy with 16 weeks of pharmacological treatment on individuals with a diagnosis of MDD and a score of 20 or greater on 17-HDRS [52]. A subanalysis was conducted using data from several prior antidepressant trials. Among those with two or more prior antidepressant trials lasting at least 4 weeks, 15 participants received cognitive therapy, while 29 participants were assigned to the pharmacotherapy group. In the pharmacotherapy group, paroxetine treatment was initiated at 10–20 mg daily for the first week and subsequently increased to a maximum dose of 50 mg daily by week six of therapy. Paroxetine non-responders received additional augmentation therapy with lithium carbonate and/or desipramine after week 8. At post-therapy, depressive symptoms measured with the HDRS were not significantly different between groups (SMD = −0.46, CI = −1.09; 0.17). The quality of evidence was graded as moderate.

A small pilot study included 12 participants experiencing a depressive episode and with a minimum score of 20 on the HDRS. All had previously failed to respond to antidepressants and any other form of therapy. This study compared 26 to 36 sessions of Cognitive Therapy (*n* = 6) with a waiting list (*n* = 6) [53]. In the Cognitive Therapy group, a significant person currently living with the participant also took part in therapy sessions. In both groups, participants were monitored by a psychiatrist throughout the treatment period. They received a subclinical dose (25 mg per day) of a tricyclic antidepressant that they were already taking. Those taking no medication at the start of the program were prescribed 25 mg of imipramine per day. At post-therapy, depressive symptoms measured using the Wakefield Depression Scale (SMD = −0.67, CI = −1.83; 0.49) and the HDRS (SMD = −0.52, CI = −1.67; 0.63) were not significantly different between groups. In addition, there was no significant difference between groups in social anxiety (SMD = −0.21, −1.34; 0.93), assertiveness (SMD = −0.22, CI = −1.35; 0.92), as well as adjustment and social behavior (SMD = −0.44, CI = −1.58; 0.71). The quality of evidence was graded as low.

At post-therapy, the pooled analysis of these two studies, involving 56 participants, showed moderate significant difference in depressive symptoms (SMD = −0.51, CI = −1.01; −0.01) between the Cognitive Therapy group and the control intervention. There was no heterogeneity between studies (I^2^ = 0.00%).

#### 3.3.4. Long-Term Psychoanalytic Psychotherapy

An RCT was conducted to compare 60 sessions of Long-Term Psychoanalytic Psychotherapy in addition to TAU (*n* = 67) with TAU alone (*n* = 62) [54]. The TAU group received care as determined by the referring practitioner or through referrals to specialized services, which could include prescribed medication, but excluded Psychoanalytic Psychotherapy. In the Long-Term Psychoanalytic Psychotherapy group, the participants could not undertake any form of short-term psychological therapy according to National Institute for Clinical Excellence guidelines. Participants included had a diagnosis of MDD with a minimum duration of two years of the current depressive episode, a minimum score of 14 on 17-HDRS and of 21 on the BDI-II, and at least two failed treatment attempts. HDRS scores assessing depressive symptoms showed no significant difference between the two groups at any time point (i.e., post-therapy, 6-, 12-, and 24-month follow-up). A small decrease in BDI-II scores was observed at post-therapy (SMD = −0.42, CI = −0.82; −0.02) and 6-month follow-up (SMD = −0.50, CI = −0.91; −0.09), and a moderate decrease at 24-month follow-up (24-month: SMD = −0.73, CI = −1.15; −0.31) in favor of Long-Term Psychoanalytic Psychotherapy. Compared with the control group, the Long-Term Psychoanalytic Psychotherapy group displayed significant improvements in social functioning at all time points (SMD ranging from 0.49 to 0.69), fewer well-being deficits at 24-month follow-up (SMD = −0.66, CI = −1.08; −0.24), and a better quality of life at 6-month (SMD = 0.57, CI = 0.16; 0.98) and 24-month follow-ups (SMD = 0.68, CI = 0.27; 1.11). In both groups, the average number of medications increased from two to five during the study, with no significant difference between groups. The quality of evidence was graded as moderate-to-high.

#### 3.3.5. Group Compassion-Focused Therapy

A small pilot RCT was conducted to assess the efficacy of 12 sessions of a group compassion-focused therapy for 4–6 individuals (*n* = 9), compared to TAU (*n* = 7) [55]. In addition to a primary diagnosis of MDD (82%) or dysthymia (18%), participants were required to have at least a moderate level of depressive symptoms and to be refractory to two selective serotonin reuptake inhibitors. The TAU group continued to receive their regular medical appointments and may have participated in rehabilitation day care programs. Still, they were asked to refrain from receiving psychological interventions such as counseling or psychotherapy during the study. At post-therapy, while GRID-HDRS scores assessing depression symptoms did not differ between groups (SMD = −0.74, CI = −1.76; 0.28), BDI-II scores significantly decreased in compassion-focused therapy (SMD = −1.59; CI = −2.72; −0.46). No significant difference was observed between the groups in self-compassion (SMD = 0.33, CI = −0.66; 1.33). A large effect was found in favor of group compassion-focused therapy in compassionate engagement both for self (SMD = 1.48, CI = 0.37; 2.60) and for others (SMD = 1.20, CI = 0.13; 2.27); no effect was found for compassionate engagement from others (SMD = 0.30, CI = −0.69; 1.29). The evidence was graded as low-to-moderate quality.

#### 3.3.6. Trauma-Focused Cognitive Behavioral Therapy

A small pilot RCT was conducted to compare 24 sessions of Trauma-Focused Cognitive Behavioral Therapy (*n* = 10) to eye movement desensitization and reprocessing (*n* = 12) [56]. Both groups received drug TAU, and adjustments were permitted based on the clinical judgment of the treating physicians. Inclusion criteria were a diagnosis of MDD, a history of at least three lifetime traumatic events, failure to respond to two or more adequate trials of antidepressants from different classes, and an adequate trial of a tricyclic antidepressant. At the post-therapy and 1-month follow-up, there was no difference between the two groups in depressive symptoms measured using the MADRS (SMD = 0.01, CI = −0.83; 0.85; SMD = −0.31, CI = −1.15; 0.54) and the BDI-II (SMD = −0.18, CI = −1.02; 0.66; SMD = −0.33, CI = −1.17; 0.52) in scores of anxiety (SMD = −0.33, CI = −1.18; 0.51; SMD = −0.25, CI = −1.09; 0.59), sleep quality (SMD = −0.26, CI = −1.11; 0.58; SMD = −0.17, CI = −1.01; 0.67), and psychosocial functioning (SMD = −0.08, CI = −0.91; 0.76; SMD = −0.28, CI = −1.12; 0.56). The quality of evidence was graded as moderate.

#### 3.3.7. Group-Based Interpersonal Psychotherapy and Occupational Therapy

A randomized trial was conducted to compare 16 sessions of Group-Based Interpersonal Psychotherapy and Occupational Therapy plus medication management (augmentation and combination strategies) (*n* = 34) with TAU (*n* = 30) [58]. In the TAU condition, participants received treatment using available community services. Participants included had a diagnosis of chronic MDD, dysthymic disorder with superimposed MDD (double depression), or MDD in partial remission, had an episode duration of two years or more, and had at least moderate severity of depressive symptoms. Participants had a history of a mean of 2.9 (SD ± 1.0) failed medication trials, and the majority (85.9%) had previously undergone psychotherapy. At post-therapy, no significant differences were observed in depressive symptoms measured with the de BDI-II (SMD = −0.29, CI = −0.82; 0.25) and in common psychiatric symptoms (i.e., somatization, obsessive-compulsive, interpersonal sensitivity, depression, anxiety) (SMD = −0.23, CI = −0.77; 0.30). The quality of evidence was graded as moderate.

#### 3.3.8. Body-Oriented Psychological Therapy

An RCT was conducted to compare 20 sessions of Body-Oriented Psychological Therapy (*n* = 12) to a waiting list (*n* = 10) [57]. Both groups received TAU, which consisted of ongoing antidepressant medication and outpatient clinical management. Participants had an MDD diagnosis, a duration of the current episode of depression of more than two years, and a total score of ≥20 on HDRS, and had failed at least two antidepressant trials and one psychological therapy before the study. At post-therapy, a large significant decrease was found in depressive symptoms measured with the HDRS (SMD = −0.92, CI = −1.80; −0.04) in the Body-Oriented Psychological Therapy group compared with the control group. No significant difference was observed between groups in quality of life (SMD = 0.28, CI = −0.57; 1.12) and self-esteem (SMD = 0.34, CI = −0.52; 1.20). The authors reported no clinically relevant medication change (i.e., drug change within four weeks before entering the study and/or more than 30% dose increase) and/or other psychological treatments. The quality of evidence was graded as moderate.

## 4. Discussion

To our knowledge, this is the first systematic review and meta-analysis assessing the efficacy of psychotherapy in TRD, which is defined as the failure of at least two treatment trials involving either pharmacotherapy or psychotherapy. In summary, when pooling all kinds of psychotherapies, our meta-analysis revealed a small but significant effect on depressive symptoms (SMD = −0.49, CI = −0.63; −0.34) in favor of the psychological intervention compared to the control group. There is currently no clear consensus regarding the effect size required to determine clinical significance. One study proposed an effect size of SMD = 0.24 as a preliminary threshold for clinical relevance in the treatment of depression [59], while another suggested that effect sizes below d = 0.5 should be considered clinically irrelevant [60]. Although psychotherapy demonstrates statistically significant effects in TRD, the clinical meaningfulness of these effects remains uncertain. Future research should place greater emphasis on evaluating the clinical significance of the outcome. When possible, separate meta-analyses were conducted by type of psychological intervention. MBCT, CBT, and Cognitive Therapy showed a moderate reduction in depressive symptoms compared to active control. These three psychotherapies may share overlapping therapeutic techniques (e.g., identifying cognitive distortions and ruminative thinking patterns). Moreover, the effect sizes associated with these psychotherapies appear to be of similar magnitude. Due to the limited number of studies available (i.e., only one study was identified for each of the treatment modalities), meta-analyses could not be conducted for other types of psychotherapy. Specifically, Trauma-Focused Cognitive Behavioral Therapy and Group-Based Interpersonal Psychotherapy and Occupational Therapy did not show statistically significant differences compared to active control conditions on depressive symptoms. A large reduction in depressive symptoms was found for Body-Oriented Psychological Therapy compared to an inactive control. Finally, Long-term Psychoanalytic Psychotherapy and Group Compassion-Focused Therapy have mixed results depending on the type of questionnaire used for assessing depressive symptoms. Moreover, most psychotherapies did not show significant effects on secondary outcomes (i.e., rumination, self-compassion, anxiety, and self-esteem) compared to the control interventions. The lack of significant effects on certain secondary outcomes may be partly explained by limited statistical power, as several included studies had small sample sizes. It is also possible that these psychotherapeutic interventions are less effective for these specific outcomes. Future studies with larger samples and targeted outcome measures are needed to clarify these findings.

In comparison, a meta-analysis of 143 studies in depression without specific criteria for treatment resistance showed that psychotherapy had a larger effect (g = −0.66, CI = −0.78; −0.53) compared to TAU [26]. Accordingly, the efficacy of psychotherapeutic interventions appears to be slightly lower in TRD relative to those with non-treatment-resistant depression. However, as the number of studies is not comparable, it is currently not possible to draw conclusions [27,61]. However, one notable finding was the diversity of countries conducting studies, reflecting a widespread global interest in treating TRD, and allowing for the inclusion of diverse cultural perspectives in the research. While this geographic diversity strengthens the generalizability of findings by encompassing a wide range of cultural and healthcare contexts, it also introduces variability that may influence treatment outcomes. A previous meta-analysis on psychotherapy for depression found a significant regional difference in effect sizes (*p* < 0.001), with the lowest effect sizes reported in North America, Europe, and Australia, and the highest in East Asia, South Asia and the Middle East, and North Africa [62].

Although this meta-analysis is clinically relevant, its findings should be interpreted with caution due to several limitations. Firstly, the small number of studies included in this meta-analysis constitutes an important methodological limitation. It limits the robustness of the conclusions that can be drawn. Therefore, it was not possible to conduct meta-analyses by type of psychotherapy for all treatment modalities or by type of control group (i.e., waiting list, TAU, other psychotherapy), which may have led to either underestimation or overestimation of the findings. Previous meta-analyses showed that effect sizes are generally smaller when interventions are compared to active control groups (such as pharmacotherapy or psychotherapy) rather than to TAU or waiting list control groups [63]. Thus, heterogeneity in control conditions across studies may have biased the aggregated outcomes. Furthermore, future studies should include comparisons between psychotherapy and emerging treatments for TRD, such as electroconvulsive therapy, ketamine, and virtual reality-based psychotherapy, to better position psychotherapy within the broader treatment landscape and identify optimal, patient-centered care pathways. Additionally, it was not possible to determine whether one psychotherapy is more effective than another, as no study directly compared different kinds of psychotherapy. A meta-analysis on the response rates in depression without criteria for treatment resistance observed that different psychotherapy modalities (i.e., CBT, Behavioral Activation, Interpersonal Psychotherapy, Problem-Solving Therapy) seemed to have a comparable effect [61]. Nevertheless, all these points underscore the need for further high-quality studies to build a stronger evidence base, particularly for TRD to at least two treatment trials. Indeed, research on TRD is comparatively scarce relative to the larger body of studies on depression without criteria for resistance to treatment [26,61,64]. Therefore, increased personalized research targeting this specific population is necessary. At present, it would be premature to assess the potential efficacy of specific components of psychotherapies. However, as more studies become available, it will be necessary to examine these components in greater detail to identify the most effective therapeutic elements for treating TRD. It would also be of interest, once more studies become available, to explore whether a dose–response relationship exists between the number of sessions and the effectiveness of psychotherapies. Among the included studies, none reported patient-reported outcomes. Future research should incorporate these measures to better capture the patient’s perspective.

Secondly, due to the limited number of studies included, it was not possible to perform sub-analyses based on different characteristics of the sample (e.g., criteria for TRD, TRD severity, sex, comorbidity). The sample was characterised by a higher proportion of women (65%) than men. This is consistent with the literature, which shows that depression is two times more prevalent in women and has significantly higher treatment-seeking rates [65,66,67]. Sex does not appear to be a significant predictive factor in the therapeutic response to depression [68,69]. However, this sex imbalance may limit the external validity of the findings, particularly regarding their applicability to males with TRD. A dedicated analysis would be relevant to determine whether this holds specifically in the context of TRD. In addition, only one study excluded all comorbidities (e.g., anxiety disorder, personality disorder). Our findings may be underestimated, as comorbidity appears to reduce the effectiveness of psychotherapy relative to what might be observed in a more diagnostically homogeneous TRD population [70]. Thus, the presence of comorbidities may obscure treatment-specific effects and complicate the identification of optimal therapeutic strategies for more homogeneous TR-MDD subgroups. In addition, comorbidities may introduce greater heterogeneity into study samples, potentially increasing variability in outcomes. However, as comorbidity is highly prevalent in individuals with MDD, this allowed a better representation of the population with a depressive disorder [71,72,73]. Indeed, the inclusion of individuals with comorbidities enhances the generalizability of the results to typical clinical settings, where strict diagnostic exclusions are rarely feasible. Regarding the inclusion criterion for TRD, which requires at least two courses of treatment, the lack of consensus on its definition and, particularly, what constitutes an adequate dose and duration, results in heterogeneity across populations enrolled in clinical trials and observed in real-world practice [15,74]. While this variability may limit the interpretation of findings, it may also better reflect the heterogeneity observed in real-world clinical practice [74]. All the limitations discussed may affect the extent to which the findings can be generalized. Thirdly, it was not possible to assess the medium- and long-term effectiveness of psychotherapy, as only two studies analyzed the effect beyond a 6-month follow-up [50,54]. Consequently, the sustained effects of psychotherapy cannot be determined. This highlights the need for future trials to incorporate longer follow-up periods to evaluate the durability of treatment effects better.

Fourthly, although concurrent pharmacotherapy could represent a potential confounding factor on the independent effect of psychotherapies, the majority of studies controlled for this by assessing medication changes during the trials and allowing pharmacotherapy use in both groups. Furthermore, combined treatment approaches (psychotherapy and pharmacotherapy) are recommended in current guidelines for the management of TRD and represent the typical trajectory of care for this population [11,23,24,25,75]. Consequently, the sample provides a more representative depiction of individuals with TRD.

Fifth, restricting the inclusion to studies published in French and English may have introduced a potential language bias, as relevant studies in other languages may have been excluded.

Finally, findings were mostly evaluated as having moderate-quality evidence. However, it is important to note that the quality of evidence varied considerably, ranging from very low quality in quasi-experimental trials to moderate-to-high quality in single-blind RCTs. Several factors contributed to the lower quality of evidence across trials, including the absence of blinding and controlled randomization procedures, small sample sizes that may lead to limited statistical power and the generalizability of the findings, and a lack of long-term follow-up. This is critical, as an RCT with methodological limitations is insufficient to support evidence-based practice. Therefore, the quality of included studies must be carefully considered when interpreting the efficacy of interventions. To overcome all of these limitations, further studies are needed with larger sample sizes, longer follow-up periods, and comparison to other psychotherapies. The literature on this topic remains in its early stages, highlighting the clear need for further research that generates high-quality evidence using gold-standard methodologies, including adequately powered sample sizes to detect treatment superiority and follow-up periods exceeding six months to assess the durability of psychotherapeutic effects [76] and the generalizability of the findings. Future research should explore the feasibility and effectiveness of implementing psychotherapies for TRD in real-world clinical settings. This includes evaluating potential barriers (e.g., accessibility, clinician training, patient adherence) as well as facilitators (e.g., acceptability, integration into multidisciplinary care). Among the studies included in this meta-analysis, cost-effectiveness evaluations were available for CBT and long-term psychoanalytic psychotherapy. Augmented CBT for TRD has been shown to be cost-effective for patients currently experiencing moderate-to-severe symptoms, both in secondary mental health care and in primary care settings [77,78]. In contrast, long-term psychoanalytic psychotherapy was not found to be cost-effective when compared with TAU [79]. Further cost-effectiveness studies are needed to assess other psychotherapy modalities for TRD.

## 5. Conclusions

Given the high prevalence of individuals with TRD who have undergone at least two treatments, and considering that TRD is a leading cause of disability and societal economic burden, prioritizing further research into effective treatments for TRD are essential. This systematic review and meta-analysis aim to summarize the literature on the efficacy of psychotherapies for TRD. However, due to the number of study limitations, there is currently limited evidence available about the effectiveness of psychotherapy for depressive symptoms in TRD. When pooling all the psychotherapy studies, the meta-analysis showed a small to moderate effect compared to control interventions (mainly TAU). Ultimately, further research on psychotherapies is necessary to enhance treatment strategies for this complex population. New avenues have also been emerging in the field for MDD, such as the use of virtual reality personalized for each patient, which, to our knowledge, has not yet been investigated explicitly in TRD. Standard treatments for depression may be insufficient for TRD, which could necessitate the implementation of personalized medicine approaches.

## Data Availability

All relevant data are within the manuscript and its Supporting Information Files.

## Supplementary Materials

The following supporting information can be downloaded at: https://www.mdpi.com/article/10.3390/jpm15080338/s1, Table S1. PRISMA Checklist; Table S2. Electronic search strategy for the systematic review conducted; Table S3. Details of the retrieved studies included.
