# Supplementary material for: Effectiveness of Psychological Therapy for Treatment-Resistant Depression in Adults: A Systematic Review and Meta-Analysis
Source: J Pers Med. 2025 Aug 1;15(8):338. doi: 10.3390/jpm15080338 (PMC12387601; doi:10.3390/jpm15080338)
Supplement: Supplementary file 1 [file jpm-15-00338-s001.zip › jpm-3752833-supplementary.pdf]

Supplementary Material

Efficacity of psychological therapy for treatment-resistant depression in adults: A systematic review and meta-analysis

Table S1. PRISMA Checklist.
Table S2. Electronic search strategy for the systematic review conducted.
Table S3. Details of the retrieved studies included.

Table S1. PRISMA Checklist.

Section and Topic	Item #	Checklist item	Location where item is reported
**TITLE**			
Title	1	Identify the report as a systematic review.	1
**ABSTRACT**			
Abstract	2	See the PRISMA 2020 for Abstracts checklist.	1
**INTRODUCTION**			
Rationale	3	Describe the rationale for the review in the context of existing knowledge.	2-3
Objectives	4	Provide an explicit statement of the objective(s) or question(s) the review addresses.	3
**METHODS**			
Eligibility criteria	5	Specify the inclusion and exclusion criteria for the review and how studies were grouped for the syntheses.	3
Information sources	6	Specify all databases, registers, websites, organisations, reference lists and other sources searched or consulted to identify studies. Specify the date when each source was last searched or consulted.	3
Search strategy	7	Present the full search strategies for all databases, registers and websites, including any filters and limits used.	3
Selection process	8	Specify the methods used to decide whether a study met the inclusion criteria of the review, including how many reviewers screened each record and each report retrieved, whether they worked independently, and if applicable, details of automation tools used in the process.	3-4
Data collection process	9	Specify the methods used to collect data from reports, including how many reviewers collected data from each report, whether they worked independently, any processes for obtaining or confirming data from study investigators, and if applicable, details of automation tools used in the process.	4
Data items	10a	List and define all outcomes for which data were sought. Specify whether all results that were compatible with each outcome domain in each study were sought (e.g. for all measures, time points, analyses), and if not, the methods used to decide which results to collect.	4
	10b	List and define all other variables for which data were sought (e.g. participant and intervention characteristics, funding sources). Describe any assumptions made about any missing or unclear information.	4
Study risk of bias assessment	11	Specify the methods used to assess risk of bias in the included studies, including details of the tool(s) used, how many reviewers assessed each study and whether they worked independently, and if applicable, details of automation tools used in the process.	4
Effect measures	12	Specify for each outcome the effect measure(s) (e.g. risk ratio, mean difference) used in the synthesis or presentation of results.	4
Synthesis methods	13a	Describe the processes used to decide which studies were eligible for each synthesis (e.g. tabulating the study intervention characteristics and comparing against the planned groups for each synthesis (item #5)).	4
	13b	Describe any methods required to prepare the data for presentation or synthesis, such as handling of missing summary statistics, or data conversions.	4
	13c	Describe any methods used to tabulate or visually display results of individual studies and syntheses.	4
	13d	Describe any methods used to synthesize results and provide a rationale for the choice(s). If meta-analysis was performed, describe the model(s), method(s) to identify the presence and extent of statistical heterogeneity, and software package(s) used.	4

Section and Topic	Item #	Checklist item	Location where item is reported
	13e	Describe any methods used to explore possible causes of heterogeneity among study results (e.g. subgroup analysis, meta-regression).	4
	13f	Describe any sensitivity analyses conducted to assess robustness of the synthesized results.	4
Reporting bias assessment	14	Describe any methods used to assess risk of bias due to missing results in a synthesis (arising from reporting biases).	4
Certainty assessment	15	Describe any methods used to assess certainty (or confidence) in the body of evidence for an outcome.	4
**RESULTS**			
Study selection	16a	Describe the results of the search and selection process, from the number of records identified in the search to the number of studies included in the review, ideally using a flow diagram.	4-5
	16b	Cite studies that might appear to meet the inclusion criteria, but which were excluded, and explain why they were excluded.	NA
Study characteristics	17	Cite each included study and present its characteristics.	5-11
Risk of bias in studies	18	Present assessments of risk of bias for each included study.	5-11
Results of individual studies	19	For all outcomes, present, for each study: (a) summary statistics for each group (where appropriate) and (b) an effect estimate and its precision (e.g. confidence/credible interval), ideally using structured tables or plots.	5-11
Results of syntheses	20a	For each synthesis, briefly summarise the characteristics and risk of bias among contributing studies.	5-11
	20b	Present results of all statistical syntheses conducted. If meta-analysis was done, present for each the summary estimate and its precision (e.g. confidence/credible interval) and measures of statistical heterogeneity. If comparing groups, describe the direction of the effect.	5-11
	20c	Present results of all investigations of possible causes of heterogeneity among study results.	5-11
	20d	Present results of all sensitivity analyses conducted to assess the robustness of the synthesized results.	NA
Reporting biases	21	Present assessments of risk of bias due to missing results (arising from reporting biases) for each synthesis assessed.	5-11
Certainty of evidence	22	Present assessments of certainty (or confidence) in the body of evidence for each outcome assessed.	5-11
**DISCUSSION**			
Discussion	23a	Provide a general interpretation of the results in the context of other evidence.	11
	23b	Discuss any limitations of the evidence included in the review.	11-13
	23c	Discuss any limitations of the review processes used.	11-13
	23d	Discuss implications of the results for practice, policy, and future research.	11-13
**OTHER INFORMATION**			
Registration and protocol	24a	Provide registration information for the review, including register name and registration number, or state that the review was not registered.	NA
	24b	Indicate where the review protocol can be accessed, or state that a protocol was not prepared.	NA
	24c	Describe and explain any amendments to information provided at registration or in the protocol.	NA
Support	25	Describe sources of financial or non-financial support for the review, and the role of the funders or sponsors in the review.	14

Section and Topic	Item #	Checklist item	Location where item is reported
Competing interests	26	Declare any competing interests of review authors.	14
Availability of data, code and other materials	27	Report which of the following are publicly available and where they can be found: template data collection forms; data extracted from included studies; data used for all analyses; analytic code; any other materials used in the review.	14

From: Page MJ, McKenzie JE, Bossuyt PM, Boutron I, Hoffmann TC, Mulrow CD, et al. The PRISMA 2020 statement: an updated guideline for reporting systematic reviews. BMJ 2021;372:n71. doi: 10.1136/bmj.n71. This work is licensed under CC BY 4.0. To view a copy of this license, visit https://creativecommons.org/licenses/by/4.0/

Table S2. Electronic search strategy used for the present meta-review.

Database; Search	Search terms
**PubMed**; k= 3 550	(depress* [Title/Abstract] OR "depressive disorder" [Title/Abstract] OR "major depress* [Title/Abstract]) AND ("Treatment-Resistant" [Title/Abstract] OR "partial remission" [Title/Abstract] OR "partial response" [Title/Abstract] OR "persistent" [Title/Abstract] OR "refractor*" [Title/Abstract] OR "treatment resistant"[Title/Abstract] OR resistan* [Title/Abstract] OR refractor* [Title/Abstract] OR non-respon* [Title/Abstract] OR nonrespon* [Title/Abstract] OR un-respon* [Title/Abstract] OR unrespon* [Title/Abstract] OR TRD [Title/Abstract] OR fail*[Title/Abstract] OR inadequate [Title/Abstract]) AND (psychotherapy* [Title/Abstract] OR intervention [Title/Abstract] OR "Psychosocial treatment" [Title/Abstract] OR "Psychological treatment" [Title/Abstract] OR therap*[Title/Abstract])
**PsycINFO**; k= 1 208	Abstract: (depress* OR "depressive disorder" OR "major depress*") AND Abstract: ("Treatment-Resistant" OR Abstract: "partial remission" OR "partial response" OR "persistent" OR "refractor*" OR "treatment resistant" OR resistan* OR refractor* OR non-respon* OR nonrespon* OR un-respon* OR unrespon* OR TRD OR fail* OR anadequate) AND Abstract: (psychotherapy* OR intervention OR "Psychosocial treatment" OR "Psychological treatment" OR therap*)
**Web of Science**; k= 3 120	(AB=(depress* OR "depressive disorder" OR "major depress*")) AND (AB=("Treatment-Resistant" OR Abstract: "partial remission" OR "partial response" OR "persistent" OR "refractor*" OR "treatment resistant" OR resistan* OR refractor* OR non-respon* OR nonrespon* OR un-respon* OR unrespon* OR TRD OR fail* OR inadequate)) AND (AB=(psychotherapy* OR intervention OR "Psychosocial treatment" OR "Psychological treatment" OR therap*)
**Cochrane**; k= 264	(depress* OR "depressive disorder" OR "major depress*") AND ("Treatment-Resistant" OR "partial remission" OR "partial response" OR "persistent" OR "refractor*" OR "treatment resistant" OR resistan* OR refractor* OR non-respon* OR nonrespon* OR un-respon* OR unrespon* OR TRD OR fail* OR inadequate) AND (psychotherapy* OR intervention OR "Psychosocial treatment" OR "Psychological treatment" OR therap*)

Note. A search in Google Scholar with the exact keywords and cross-referencing enabled the finding of an additional 38 articles.

Table S3. Details of the retrieved studies.

Authors, years	Design of studies Studies countries	Description of sample Sample size (% men) Inclusion criteria Comorbidity	Definition of treatment-resistant reported by studies	Intervention group Number of sessions Training and fidelity of therapists	Control group	Results	Moderators	Quality of evidence
Mindfulness-based cognitive therapy								
Cladder-Micus et al. 2018	RCT (no blind assessors)<br><br>Netherlands	96 (38%) (44 MBCT + TAU, 52 TAU)<br><br>**Inclusion criteria**<br>- current depressive episode had lasted at least 12 months<br>- score ≥21 on Inventory of Depressive Symptomatology-Self-Report<br><br>**Comorbidity**<br>- anxiety disorder (47%)	At least one adequate trial of antidepressant medication during the current episode (defined as appropriate doses of antidepressant medication for ≥4 weeks or patient's refusal to use medication contrary to the advice of a psychiatrist), and (e) previous psychological treatment during the current episode (defined as ≥10 sessions of CBT or IPT or < 10 sessions if discontinued because of patient's withdrawal).	8 sessions<br><br>The intervention was provided by mindfulness trainers who were highly experienced in working with depressed patients and had completed a 2-year postgraduate mindfulness teacher training. Teacher competence and adherence were assessed with the Mindfulness-Based Interventions-Teaching Assessment Criteria based on two video-recorded sessions per trainer	TAU (naturalistic condition consisting of mental health care for depression, including antidepressant medication, psychological treatment excluding mindfulness-based, support by a psychiatric nurse, or day hospital treatment)	***Depressive symptoms***<br>Inventory of Depressive Symptomatology-Self Report<br>**Post: SMD=-0.43, CI=-0.83; -0.02 ***<br><br>***Mindfulness skills***<br>5-Facet Mindfulness Questionnaire<br>**Post: SMD=0.67, CI=0.24; 1.10 ***<br><br>***Self-compassion***<br>Self-Compassion Scale<br>**Post: SMD=0.52, CI=0.09; 0.95 ***<br><br>***Rumination***<br>Ruminative Response Scale<br>Post: SMD=-0.38, CI=-0.80; 0.05 *<br><br>***Quality of life***<br>World Health Organization Quality of Life scale<br>**Post: SMD=0.45, CI=0.03; 0.88 ***	Gender, age, childhood trauma, number of previous episodes, duration of current depressive episode, treatment resistance, baseline levels of depressive symptoms, rumination, mindfulness skills, and self-compassion	Moderate
Eisendrath et al. 2016	RCT (blind assessors)<br><br>USA	131 (27.7%) (67 MBCT, 64 health enhancement program)<br><br>**Inclusion criteria**<br>- diagnosis of MDD<br>- score ≥14 on 17-Hamilton Depression Rating Scale<br><br>**Comorbidity**<br>- anxiety disorder (60.0%), eating disorder (12.7%), and	Taking antidepressant medications with evidence of two or more adequate trials prescribed during the current episode	8 sessions<br><br>The intervention were provided by extensively trained in their respective techniques, having at least 3 years of experience in their respective components.<br>All group sessions were audio recorded, and three recordings per group were randomly selected and reviewed	Health-enhancement program (Course content focused on aerobic exercise, functional movement, music therapy, and dietary education. Participants were asked to do specific exercises (e.g., walking/stretching/agility training), receive nutritional education, and participate in musical activities such as drumming or song writing).	***Depressive symptoms***<br>Hamilton Depression Rating Scale<br>**Post: SMD=-0.58, CI=-0.93; -0.23 ***<br><br>***Mindfulness skills***<br>5-Facet Mindfulness Questionnaire<br>Post: SMD=0.15, CI=-0.01; 0.30 *<br><br>***Self-compassion***<br>Self-Compassion Scale<br>Post : SMD=0.14, CI=-0.20; 0.49 *<br><br>***Rumination***<br>Ruminative Response Scale<br>Post : SMD=0.05, CI=-0.39; 0.29 *<br><br>***Experiential avoidance***	Ethnicity, minority, socio-economic status, age of depression onset, number of lifetime depressive episodes, co-morbid psychiatric illness (e.g. personality disorders), anxiety, current stress, early childhood stress, expectations and beliefs about treatment	Moderate

		- (personality disorder (16.0%)		by external and internal evaluators. The intervention fidelity was assessed through an internal UCSF reviewer and externally by an unaffiliated psychiatrist trained in MBCT.		Acceptance and Action Questionnaire<br>Post : SMD=0.00, CI=-0.34, 0.34 *		
Foroughi et al., 2020	RT<br>(no blind assessors)<br><br>Iran	18 (38.46%)<br>(9 MCBT + antidepressants, 9 antidepressants<br><br>**Inclusion criteria**<br>- diagnosis of MDD<br>- score ≥ 17 on Beck depression Inventory-II	Lack of therapeutic response to adequate doses of two antidepressants for a sufficient time	8 sessions<br><br>The intervention was conducted by a Master of Clinical Psychology, who was trained in this field, and the supervisor monitored all of the treatment sessions.	Antidepressants (initially treated with a dose of 60 mg citalopram for four to six weeks. Due to the lack of response during this period, they were given Sertraline at a maximum dose of 200 mg for four to six weeks. In addition to sertraline, bupropion was also prescribed. Anti-depressant medications were administered with appropriate dosages and durations).	***Depressive symptoms***<br>Beck Depression Inventory-II<br>**Post: SMD=-1.26, CI=-2.27; -0.25 ***<br>1-month: SMD=-0.91, CI=-1.88; 0.06 *<br>Hamilton Depression Rating Scale<br>Post: SMD=-0.43, CI=-1.36, 0.50 *<br>**1-month: SMD=-2.45, CI=-3.67; -1.23 ***<br><br>***Self-compassion***<br>Self-Compassion Scale – Short Form<br>Post: SMD=1.14, CI=-0.57; 2.86 *<br>**1-month: SMD=3.09, CI=1.73; 4.46 ***<br><br>***Rumination***<br>Ruminative Response Scale<br>**Post: SMD=-1.06, CI=-2.04; -0.07 ***<br>**1-month: SMD=-1.29, CI=-2.31; -0.28 ***<br><br>***Mindfulness skills***<br>Southampton Mindfulness Questionnaire<br>**Post: SMD=2.16, CI=1.00; 3.32 ***<br>**1-month: SMD=3.63, CI=2.12; 5.13 ***	None	Low

Cognitive behavioral therapy								
Hauksson et al. 2017	RT<br>(blind assessors)<br><br>Iceland	95 (30.5%)<br>(59 individual CBT + rehabilitation treatment,<br>36 rehabilitation treatment)<br><br>**Inclusion criteria**<br>- diagnosed with MDD (83,9%) or dysthymia (36,5%)<br><br>**Comorbidity**<br>- average of 5-6 comorbidities per participant (e.g., anxiety, psychotic, eating disorder, posttraumatic stress disorder, substance dependence, obsessive-compulsive disorder)	Failed to respond to at least two antidepressant trials of adequate doses and duration (corresponding to the equivalent of at least 20 mg of fluoxetine for at least 2 months)	12 sessions<br><br>The therapist of the intervention, including a psychiatrist, a psychologist, psychiatric nurses, occupational therapists and a social worker, had at least 1 year training in CBT and were a part of the unit's interdisciplinary clinical team. The training included education in CBT theory and case formulation, and covered topics such as Socratic questioning, teaching of basic cognitive and behavioural methods, relapse prevention, symptoms and treatment of depression and anxiety disorders, chronic pain, and chronic illness. The therapists received 1-hour weekly group supervision from a specialist in CBT to ensure treatment fidelity and to provide guidance in individual cases.	Rehabilitation treatment (psychoeducation, behavioral activation, occupational therapy, relaxation, counseling by a member of professional health or social care, and medication as needed)	***Depressive symptoms***<br>Beck Depression Inventory-II<br>**Post: SMD=-0.50, CI=-0.92; -0.08 ***<br>18-month: SMD=-0.14, CI=-0.66; 0.38 *<br><br>***Anxiety symptoms***<br>Beck Anxiety Inventory<br>Post: SMD=-0.17, CI=-0.59; 0.24 *<br>18-month: SMD=-0.13, CI=-0.65, 0.40 *<br><br>***Hopelessness***<br>Beck Hopelessness Scale<br>**Post: SMD=-0.53, CI=-0.95; -0.11 ***<br>18-month: SMD=-0.36, CI=-0.88; 0.17 *<br><br>***Automatic thoughts***<br>Automatic Thoughts Questionnaire<br>Post: SMD=-0.31, CI=-0.72; 0.11 *<br>18-month: SMD=-0.03, CI=-0.55; 0.49 *	Psychiatric diagnosis and comorbidity (i.e., major depressive episode, generalized anxiety disorder, social phobia, panic disorder, post-traumatic stress disorder, substance dependence, hypomanic episode, obsessive-compulsive disorder, psychotic disorder, bulimia, anorexia.	Moderate
		145 (23.4%)<br>(59 individual CBT + rehabilitation treatment,<br>86 group CBT + rehabilitation treatment)			Group CBT + rehabilitation treatment	***Depressive symptoms***<br>Beck Depression Inventory-II<br>Post: SMD=-0.01, CI=-0.40; 0.38 *<br>18-month: SMD=-0.03, CI=-0.42; 0.36 *<br><br>***Anxiety symptoms***<br>Beck Anxiety Inventory<br>Post: SMD=-0.02, CI=-0.41; 0.37 *<br>18-month: SMD=0.13, CI=-0.26; 0.52 *<br><br>***Hopelessness***<br>Beck Hopelessness Scale<br>Post: SMD=-0.04, CI=-0.43; 0.35 *<br>18-month: SMD=0.07, CI=-0.32; 0.46 *<br><br>***Automatic thoughts***<br>Automatic Thoughts Questionnaire<br>Post: SMD=0.00, CI=-0.39; 0.39 *<br>18-month: SMD=0.05, CI=-0.34; 0.44 *		Moderate-to-high

Wilkinson et al. 2021	RT<br>(blind assessors)<br><br>USA	28 (46.4%)<br>(14 CBT, 14 TAU)<br><br>**Inclusion criteria**<br>- diagnosis of MDD<br>- score ≥21 on 17-Hamilton Depression Rating Scale	Treatment resistance to ≥ 2 adequate courses of antidepressant medications	16 sessions<br><br>The intervention was conducted by two experienced therapists who had received prior training and certification from the Beck Institute for CBT and had participated in several prior clinical trials involving CBT	TAU (weekly or every-other-week visits with a study physician for the management of medication and adverse events)	***Depressive symptoms***<br>Montgomery-Asberg Depression Rating Scale<br>Post: SMD=-0.65, CI=-1.82; 0.55<br>Quick Inventory of Depressive Symptomatology-Self-report<br>Post: SMD=-0.71; CI=0-1.70; 0.28	None	Low-to-moderate
Cognitive therapy								
Leykin et al. 2020	RCT<br>(blind assessors)<br><br>USA	44 (NA)<br>(15 CT, 29 pharmacotherapy)<br><br>**Inclusion criteria**<br>- diagnosis of MDD<br>- score ≥ 20 on 17-Hamilton Depression Rating Scale	≥ 2 prior antidepressant trials for at least 4 weeks	20-28 sessions<br><br>The intervention was provided by three therapists at each site, with CT experience ranging from 5 to 21 years. All therapists followed standard CT as described in A. T. Beck,<br>Rush, Shaw, and Emery (1979), A.T. Beck and Freeman (1990),<br>and J. Beck (1995).	Pharmacotherapy (Paroxetine treatment was initiated with 10–20 mg daily for the 1st week and was subsequently raised to a maximum dose of 50 mg daily by Week 6 of therapy, as clinically warranted. The minimum acceptable dose of paroxetine was 20 mg daily. Paroxetine nonresponders (i.e., those having an HRSD score of 12) received additional augmentation therapy with lithium carbonate and/or desipramine after Week 8 of AD therapy)	***Depressive symptoms***<br>Hamilton Depression Rating Scale<br>Post : SMD=-0.46, CI=-1.09; 0.17 *	None	Moderate
Harpin, 1982	Pilot<br>(no blind assessors)<br><br>England	12 (58.3%)<br>(6 cognitive therapy, 6 waiting list)<br><br>**Inclusion criteria**<br>- depressive episode<br>- score ≥ 20 on 17-Hamilton Depression Rating Scale	Been unresponsive to antidepressant and any other form of therapy that might have been tried.	20 sessions<br><br>The intervention was provided by a fifth-year doctoral candidate in clinical psychology who had completed all course and practicum requirements as well as a master's degree	Waiting list	***Depressive symptoms***<br>Wakefield Depression<br>Post: SMD=-0.67, CI=-1.83; 0.49 *<br>Hamilton Depression Rating Scale<br>Post: SMD=-0.52, CI=-1.67; 0.63 *<br><br>***Social Anxiety***<br>Falloon Social Anxiety Scale<br>Post: SMD=-0.21, -1.34; 0.93 *<br><br>***Assertiveness***	None	Low

				in behavioral counseling. Supervision in the development of the treatment package and its implementation was provided by both experienced behavioral psychiatrists.		Adult Self-Expression Scale<br>Post: SMD=-0.22, CI=-1.35; 0.92 *<br><br>***Symptoms Discomfort***<br>Katz Social Adjustment Scale<br>Post: SMD=-0.44, CI=-1.58; 0.71 *<br><br>***Adjustment and social behavior***<br>Katz Social Adjustment Scale - Level of Performance of Socially Expected Activities<br>Post: SMD=-0.47, CI=-1.62; 0.67 *<br>Katz Social Adjustment Scale - Level of Expectation of Performance of Socially Expected Activities<br>Post: SMD=-0.08, CI=-1.21; 1.06 *<br>Katz Social Adjustment Scale - Level of Free-Time Activities<br>Post: SMD=-0.09, CI=-1.23; 1.04 *<br>Katz Social Adjustment Scale - Level of Satisfaction with Free-Time Activities<br><br>Post: SMD=-0.01, CI=-1.14, 1.12 *		
Long-Term Psychoanalytic Psychotherapy								
Fonagy et al. 2015	RCT (blind assessors)<br><br>United Kingdom	99 (33%)<br>(51 psychoanalytic psychotherapy + TAU, 48 TAU)<br><br>**Inclusion criteria**<br>- diagnostic of MDD<br>- minimum duration of two years of the current depressive episode<br>- score ≥ 14 on 17-Hamilton Depression Rating Scale<br>- score ≥21 on Beck Depression Inventory-II<br><br>**Comorbidity**<br>- anxiety disorder (75.2%), substance use disorder	At least two failed treatment attempts, one of which must have included treatment with an antidepressant medication and the other with either an antidepressant medication or a psychological intervention.	60 sessions<br><br>All the therapists (n=22; average years of experience: (17.45) had a mental health qualification and a training approved by the British Psychoanalytic Council. All therapy sessions were audio-recorded. Fidelity to treatment was assessed with the 100-item Psychotherapy Process Q-Sort. Three randomly selected sessions from the early, middle, and end phases of each	TAU (interventions as directed by the referring practitioner or referrals to other specialized services that might include prescribed medication but could not be psychoanalytic psychotherapy)	***Depressive symptoms***<br>Hamilton Depression Rating Scale<br>Post: SMD=-0.23, CI=-0.63; 0.16 *<br>6-month: SMD=-0.34, CI=-0.75; 0.06 *<br>12-month: SMD=-0.38, CI=-0.78; 0.02 *<br>24-month: SMD=-0.67, CI=1.10; -0.25 *<br><br>Beck Depression Inventory-II<br>**Post: SMD=-0.42, CI=-0.82; -0.02 ***<br>**6-month: SMD=-0.50, CI=-0.91; -0.09 ***<br>12-month: SMD=-0.25, CI=-0.65; 0.15 *<br>**24-month: SMD=-0.73, CI=-1.15; -0.31 ***<br><br>***Social functioning***<br>Global Assessment of Functioning<br>**Post: SMD=0.50, CI=0.10; 0.90 ***<br>**6-month: SMD=0.61, CI=0.20; 1.02 ***<br>**12-month: SMD=0.49, CI=0.09; 0.89 ***<br>**24-month: SMD=0.69, CI=0.27; 1.11 ***<br><br>***Well-being deficits***<br>Clinical Outcomes in Routine Evaluation	None	Moderate-to-high

		(18.6%), eating disorder (13.2%)		treatment were rated (183 sessions in total). Inter-rater reliability, assessed in a subsample of 90 sessions, was excellent.		Post: SMD=-0.40, CI=-0.79; 0.00 *<br>6-month: SMD=-0.37, CI=-0.78; 0.03 *<br>12-month: SMD=-0.23, CI=-0.63; 0.16 *<br>**24-month: SMD=-0.66, CI=-1.08; -0.24 ***<br><br>***Quality of life***<br>Quality of Life Enjoyment and Satisfaction Questionnaire<br>Post: SMD=0.32, CI=-0.07; 0.72 *<br>**6-month: SMD=0.57, CI=0.16; 0.98 ***<br>12-month: SMD=0.30, CI=-0.10; 0.70 *<br>**24-month: SMD=0.68, CI=0.27; 1.11 ***		

Group Compassion-Focused Therapy								
Asano et al., 2022	RCT<br>(No blind assessors)<br><br>Japan	16 (12%)<br>(9 group compassion-focused therapy + TAU,<br>7 TAU)<br><br>**Inclusion criteria**<br>- diagnosis of MDD (82%) or dysthymia (18%)<br>- score ≥20 on Beck Depression Inventory-II<br><br>**Comorbidity**<br>- (59%) have another comorbidity (i.e., anxiety, obsessive-compulsive disorder, post-traumatic stress disorder, and/or bulimia nervosa)	Refractory to two Selective Serotonin Reuptake Inhibitor treatments	12 sessions<br><br>A therapist and a co-therapist conducted the sessions. The therapist was a clinical psychologist with a Ph.D. in psychology who had trained at a three-day CFT workshop. The co-therapist was an industrial counselor. Both the therapist and co-therapist had completed a CBT training course at Chiba University. Peer supervisions were conducted once a week by the therapist and co-therapist for quality control, and the fidelity checklist was confirmed. In addition, the therapist received supervision via a video meeting system from the other CFT therapists once a month.	TAU (continued to receive their regular medical appointments and may participate in rehabilitation day care programs, but they are asked to refrain from receiving psychological interventions such as counseling or psychotherapy)	***Depressive symptoms***<br>Beck Depression Inventory-II<br>**Post: SMD=-1.59, CI=-2.72, -0.46 ***<br>GRID-Hamilton Depression Rating Scale<br>Post: SMD=-0.74, CI=-1.76; 0.28 *<br><br>Compassionate Engagement and Action Scale - For self<br>**Post: SMD=1.48, CI=0.37; 2.60 ***<br><br>Compassionate Engagement and Action Scale - For others<br>**Post: SMD=1.20, CI=0.13; 2.27 ***<br><br>Compassionate Engagement and Action Scale - From others<br>Post: SMD=0.11, CI=-0.88; 1.10 *<br><br>Fears of Compassion Scale - for others<br>Post: SMD=0.05, CI=-0.94; 1.04 *<br><br>Fears of Compassion Scale - concern from others<br>Post: SMD=0.17, CI=-0.82; 1.16 *<br><br>Fears of Compassion Scale - compassion avoidance from others<br>Post: SMD=0.30, CI=-0.69; 1.29 *<br><br>Fears of Compassion Scale - miserable with self-compassion<br>**Post: SMD=-1.80, CI=-2.97; -0.63 ***<br><br>Fears of Compassion Scale - demerit of self-compassion<br>**Post: SMD=-1.33, CI=-2.42; -0.24 ***<br><br>***Self-compassion***<br>Self-compassion Scale–Short Form<br>Post: SMD=0.33, CI=-0.66; 1.33 *	None	Low-to-moderate

Trauma-Focused Cognitive Behavioral Therapy								
Minelli et al. 2019	RCT (blind assessors)<br><br>Italy	22 (16%)<br>(10 trauma-focused CBT + drug TAU, 12 eye movement desensitization and reprocessing+ drug TAU)<br><br>**Inclusion criteria**<br>- diagnosis of MDD<br>- ≥3 traumatic events in their lifetime<br><br>**Comorbidity**<br>- psychotic symptoms (22.7%), personality disorders (68.2%), anxiety disorders (77.3%), and post-traumatic stress disorder (40.9%)	Failure to respond to ≥ 2 adequate trials of two or more different classes of antidepressants and an adequate trial of a tricyclic antidepressant drug	24 sessions<br><br>Both interventions were carried out by highly experienced psychotherapists.	Eye movement desensitization and reprocessing + drug TAU (drug treatment as usual, and adjustment were permitted based on the clinical judgment of the treating physicians)	***Depressive symptoms***<br>Montgomery–Åsberg Depression Rating Scale<br>Post: SMD=0.01, CI=-0.83; 0.85 *<br>1 month: SMD=-0.31, CI=-1.15; 0.54 *<br><br>Beck Depression Inventory-II<br>Post: SMD=-0.18, CI=-1.02; 0.66 *<br>1 month: SMD=-0.33, CI=-1.17; 0.52 *<br><br>***Anxiety symptoms***<br>Beck Anxiety Inventory<br>Post: SMD=-0.33, CI=-1.18; 0.51 *<br>1 month: SMD=-0.25, CI=-1.09; 0.59 *<br><br>***Sleep Quality***<br>Pittsburgh Sleep Quality Index<br>Post: SMD=-0.26, CI=-1.11; 0.58 *<br>1 month: SMD=-0.17, CI=-1.01; 0.67 *<br><br>***Psychosocial functioning***<br>MINI-ICF-APP<br>Post: SMD=-0.08, CI=-0.91; 0.76 *<br>1 month: SMD=-0.28, CI=-1.12; 0.56 *	baseline score of the Montgomery–Åsberg Depression Rating Scale	Moderate

Group-based Interpersonal psychotherapy and occupational therapy								
Murray et al. 2010	RT (blind assessors)<br><br>Canada	64 (85%) (34 Group-based Interpersonal psychotherapy and occupational therapy + medication management, 30 TAU)<br><br>**Inclusion criteria**<br>- diagnosis of chronic MDD, dysthymic disorder with superimposed MDD (double depression), or MDD in partial remission<br>- episode duration of ≥2 years<br>- score ≥15 on 17-Hamilton Depression Rating Scale	History of mean 2.9 (SD ± 1.0) failed medication trials, and the majority (85.9%) have previously undergone psychotherapy	16 sessions<br><br>The intervention was provided by psychiatrists trained by the project leaders, and fidelity was ensured by live observation and feedback from a senior psychotherapist	TAU (treated using available services in the community)	***Depressive symptoms***<br>Beck Depression Inventory-II<br>Post: SMD=-0.29, CI=-0.82; 0.25 *<br><br>***Common psychiatric symptoms (i.e., somatization, obsessive-compulsive, interpersonal sensitivity, depression, anxiety)***<br>Hopkins Symptom Checklist<br>Post: SMD=-0.23, CI=-0.77; 0.30 *	None	Moderate
Body Oriented Psychological Therapy								
Röhricht et al. 2013	RCT (blind assessors)<br><br>United Kingdom	22 (58%) (12 body oriented psychological therapy + TAU, 10 waiting list + TAU)<br><br>**Inclusion criteria**<br>- diagnosis of MDD with chronic depressive episode and/or chronic affective disorder (dysthymia)<br>- score ≥20 on the 21-item Hamilton Rating Scale for Depression<br>- duration of the current episode of depression of ≥2 years	Past treatments for depression of participating patients included two to eight different antidepressants and one to two courses of psychological therapy (CBT and/or Psychodynamic Psychotherapy)	20 sessions<br><br>The intervention was conduct by a part-time experienced dance movement therapist following a two-day manual training; this included the introduction of principles of the interventions, experiential learning in the form of a hands-on workshop in which therapists were introduced to the manualized treatment approach for these patients, and a	Waiting list +TAU	***Depressive symptoms***<br>Hamilton Depression Rating Scale<br>**Post: SMD=-0.92, CI=-1.80; -0.04** *<br><br>***Quality of life***<br>Manchester Short Assessment of Quality of Life<br>Post: SMD=0.28, CI=-0.57; 1.12 *<br><br>***Self-esteem***<br>Rosenberg Self-Esteem Scale<br>Post: SMD=0.34, CI=-0.52; 1.20 *	None	Moderate

				seminar on practical considerations in delivering this approach with supervised role play for the actual experience of delivering therapy sessions. The group therapy was supervised by a senior therapist in order to control for adherence to the given treatment manual (on the basis of written records and video tapes of each session) and to provide clinical supervision (after sessions 3, 8, 13 and 18 of the 20-session therapy course).				

RT; randomized trial; RCT: randomized controlled trial; TAU; treatment as usual; CBT: cognitive behavioral therapy; NA: not available; SMD: standard mean difference; MDD: major depressive disorder

* Effect sizes were calculated from primary data from the studies. Significant results are in bold.
